# 

**Published:** 1889-02-02

**Authors:** 


### CONTENTS.

**"Rod" or "Cane?"** - 275

**A Winter in Egypt** - 276

**The Doctor's Pulpit.**—"All Sorts and Conditions" of Women - 277

**A Land of Charity.** By A Colonist - 278

**The Relation of Hospitals to Medical Education** - 279

**Within the Wards.**—Wry-neck—Football-ear—A Human "Brute" - 280

**Babies.**—V. Phenomenally Large and Small Babies - 281

**Notes and News** - 282

**Everybody's Page** - 284

**Annotations.**—The Universal Cure at Last—"Physicians' Whims" or Women's Faults?—What is "Pap"? - 285

**Voyages in Vacation.**—Sweden and Stockholm - 286

**False Impressions.** By Caroline Fothergill, Author of "An Enthusiast," "A Voice in the Wilderness," etc. - 287

**Contributions by Patients to Hospitals.**—Discussion on Mr. Nelson Hardy's Paper - 288

**Scraps and Gleanings** - 290

**Amusements and Relaxation** - 290

**EXTRA SUPPLEMENT—THE NURSING MIRROR.**

**En Passant.**—A Brave Nurse—St. Thomas's Hospital—A Christmas Tree at Perth—Notes from America—District Nursing at Ashton—Massage—A Nurses' Ball—A Medical Mission Story—The British Nurses' Plans - lxix

**Notes from Australia.**—Melbourne - lxx

**A Christmas Holiday.**—The First Cottage Hospital - lxxi

**Modern Prophets on Truthfulness** - lxxi

**Everybody's Opinion** - lxxii

**Appointments, Presentations** - lxxii

**Vacancies, Notes and Queries** - lxxii

